# Immunoglobulin G responses to variant forms of *Plasmodium vivax* merozoite surface protein 9 upon natural infection in Thailand

**DOI:** 10.1038/s41598-021-82928-4

**Published:** 2021-02-05

**Authors:** Sunisa Songsaigath, Takashi Makiuchi, Chaturong Putaporntip, Urassaya Pattanawong, Napaporn Kuamsab, Hiroshi Tachibana, Somchai Jongwutiwes

**Affiliations:** 1 Department of Infectious Diseases, Tokai University School of Medicine, Isehara, Kanagawa Japan; 2 Molecular Biology of Malaria and Opportunistic Parasites Research Unit, Department of Parasitology, Faculty of Medicine, Chulalongkorn University, Bangkok, Thailand; 3 Inter-Department Program of Biomedical Sciences, Faculty of Graduate School, Chulalongkorn University, Bangkok, Thailand

**Keywords:** Parasitology, Infectious diseases, Immunology, Microbiology

## Abstract

Merozoite surface protein 9 (MSP9) constitutes a ligand complex involved in erythrocyte invasion by malarial merozoites and is a promising vaccine target. *Plasmodium vivax* MSP9 (PvMSP9) is immunogenic upon natural malaria exposure. To address whether sequence diversity in PvMSP9 among field isolates could affect natural antibody responses, the recombinant proteins representing two variants each for the N- and the C-terminal domains of PvMSP-9 were used as antigens to assess antibody reactivity among 246 *P. vivax*-infected patients’ sera from Tak and Ubon Ratchathani Provinces in Thailand. Results revealed that the seropositivity rates of IgG antibodies to the N-terminal antigens were higher than those to the C-terminal antigens (87.80% vs. 67.48%). Most seropositive sera were reactive to both variants, suggesting the presence of common epitopes. Variant-specific antibodies to the N- and the C-terminal antigens were detected in 15.85% and 16.70% of serum samples, respectively. These seropositivity rates were not significant difference between provinces. The seropositivity rates, levels and avidity of anti-PvMSP9 antibodies exhibited positive trends towards increasing malaria episodes. The IgG isotype responses to the N- and the C-terminal antigens were mainly IgG1 and IgG3. The profile of IgG responses may have implications for development of PvMSP9-based vaccine.

## Introduction

*Plasmodium vivax* attributable illness is the most geographically widespread morbidity of all human malaria with approximately 3.5 billion people in tropical and subtropical regions at risk of infection. The presence of difficult-to-treat hypnozoites responsible for chronic relapsing ailments and the emergence of chloroquine-resistant strains have made infections with *P. vivax* difficult to control and eliminate by current intervention strategies^1^. As an alternative means, vaccine development is a challenging approach to control malaria.

Proteins that play crucial roles during merozoite invasion of erythrocytes are attractive vaccine targets. Of these, a 101 kDa protein of *P. falciparum* known as acidic-basic repetitive antigen (ABRA) or merozoite surface protein 9 (PfMSP9) forms a co-ligand complex with the 19 kDa fragment of merozoite surface protein 1 (MSP1_19_) that binds to erythrocyte band 3 during the process of erythrocyte recognition/invasion^2–4^. PfMSP9 is peripherally associated with the merozoite surface and is found within the parasitophorous vacuole of *P. falciparum*^5,6^. Importantly, PfMSP9 possesses chymotrypic-like proteolytic activity akin to malarial proteases implicated in erythrocyte entry^4,6,7^ while synthetic peptides derived from this protein could bind to erythrocytes^8^. Antibodies raised against MSP9 could interfere with merozoite invasion of erythrocytes^9^ and parasite growth *in vitro*^10^. Meanwhile, immunization of mice with the *P. berghei* MSP9 homologue has conferred protection against the parasite challenge^11^. Furthermore, antibodies to *P. vivax* MSP9 (PvMSP9) were associated with protection against symptomatic infections among children in Papua New Guinea^12^, supporting the role of MSP9 as a promising vaccine candidate.

**Figure 1:**
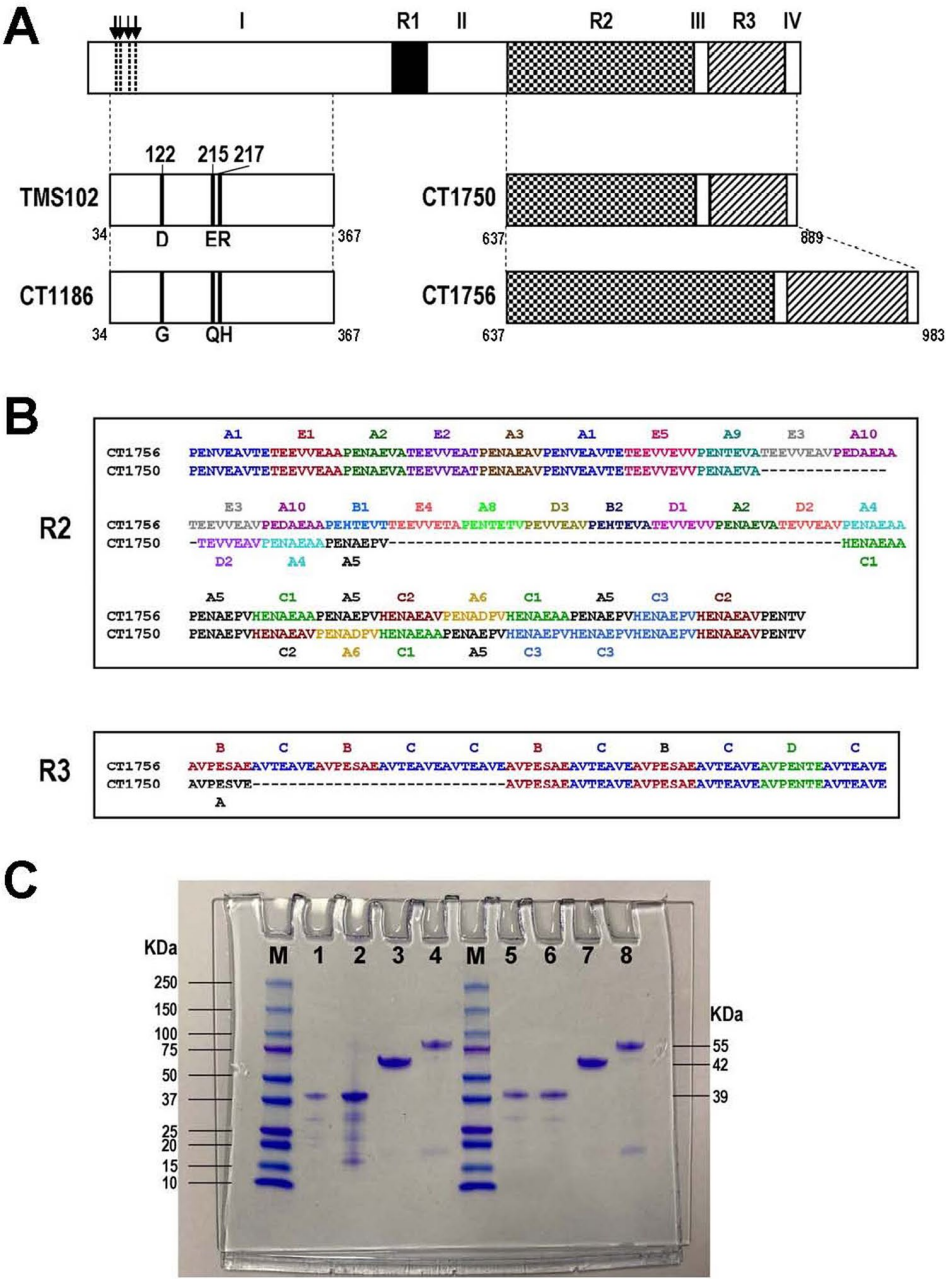
(**A**) Structure of PvMSP9 depicting conserved domains (I–III) and repeat domains (R1–R3). Approximate locations, amino acid substitutions and length of repeat domains in the recombinant proteins are shown correspondingly underneath. Four conserved cysteines at residues 49, 52, 64 and 71 are marked with downward arrows and broken vertical lines in the upper diagram. Positions of the first and the last amino acid residues for each recombinant protein are shown underneath at both ends of the schemes (**B**) Amino acid sequences of repeat domains R2 and R3 in recombinant proteins CT1750 and CT1756. The nomenclatures for the repeat units are listed above and below the sequences with corresponding colors. (**C**) Coomassie brilliant blue-stained SDS gel of purified PvMSP9 recombinant proteins. Lane M represents molecular weight marker. Lanes 1–4 are proteins TMS102, CT1186, CT1750 and CT1756, respectively, under reducing condition (with β-mercaptoethanol). Lanes 5–8 are the corresponding proteins under non-reducing condition (without β-mercaptoethanol).

Structural organization of PvMSP9 was similar to the orthologues in *P. falciparum* and other simian malaria species^13^, characterized by (1) the N-terminal signal peptide, (2) conserved cysteine residues at the N-terminal part and (3) repetitive amino acid sequences intervening conserved domains in which the locations of repeats seem to be variable across species^14^. It is noteworthy that the N-terminal conserved domain I of PvMSP9 shared 82% and 77% amino acid sequence identity with its orthologs in *P. cynomolgi* (PcyMSP9) and *P. knowlesi* (PkMSP9), respectively (Supplemental Fig. S1), rendering antigenic cross-reactivity among these malaria species^9,13^. Meanwhile, analysis of the complete PvMSP9 sequences from Thai field isolates has revealed a relatively short repeat region in the middle portion of the protein whereas two other repeat domains occupied the majority of the C-terminal region. Extensive length and sequence diversity occurred mainly in the repeat domains of PvMSP9 among field isolates^15^ (Fig. 1).

Like PfMSP9, both N- and C-terminal domains of PvMSP9 were immunogenic upon natural *P. vivax* infection^16–18^. To further address whether sequence variation in PvMSP9 could affect the profile of IgG antibody responses during natural *P. vivax* infection, we analyzed serum samples from malaria patients in Thailand against recombinant peptides derived from the N- and the C-terminal variants of this protein. Results revealed that most sera seem to recognize common or cross-reactive epitopes while some patients developed variant-specific antibodies. The majority of anti-PvMSP9 IgG antibodies were cytophilic isotypes while the levels and avidity of antibodies seems to be correlated with previous malaria episodes.

## Results

### Recombinant PvMSP9 proteins

The sodium dodecyl sulfate polyacrylamide gel electrophoresis (SDS-PAGE) of purified recombinant proteins from the N-terminal part of PvMSP-9 (TMS102 and CT1186) measured approximately 39 kDa, corresponding to the expected molecular mass. No shift in the migration pattern was observed for the N-terminal antigens under reducing and nonreducing SDS-PAGE, suggesting that the 4 cysteine residues in the recombinant proteins neither formed intermolecular nor intramolecular disulfide bridges (Fig. 1C and Supplemental Method S1). On the other hand, both C-terminal recombinant peptides from isolates CT1756 and CT1750 displayed aberrant migration on electrophoresis at approximately 42 and 55 kDa which were greater than the expected calculated molecular masses of 27 and 37 kDa, respectively (Fig. 1C and Supplemental Method S1). Anomalous migration of the C-terminal peptides in reduced electrophoresis could stem from the predominant amino acids in the repeat regions of these proteins in which the calculated pIs for CT1750 and CT1756 peptides were 3.80 and 3.62, respectively. Based on Guan and colleagues’ formula: *y* = 276.5*x−*31.33 when *x* and *y* represent the percentage of acidic amino acids and the average difference of molecular weight per amino acid^19^, the migration of CT1750 and CT1756 peptides on SDS-PAGE was calculated to be 39.2 and 54.2 kDa which were close to the observed migration of the C-terminal peptides in this study.

### Prevalence of anti-IgG antibodies to PvMSP9 antigens

**Figure 2:**
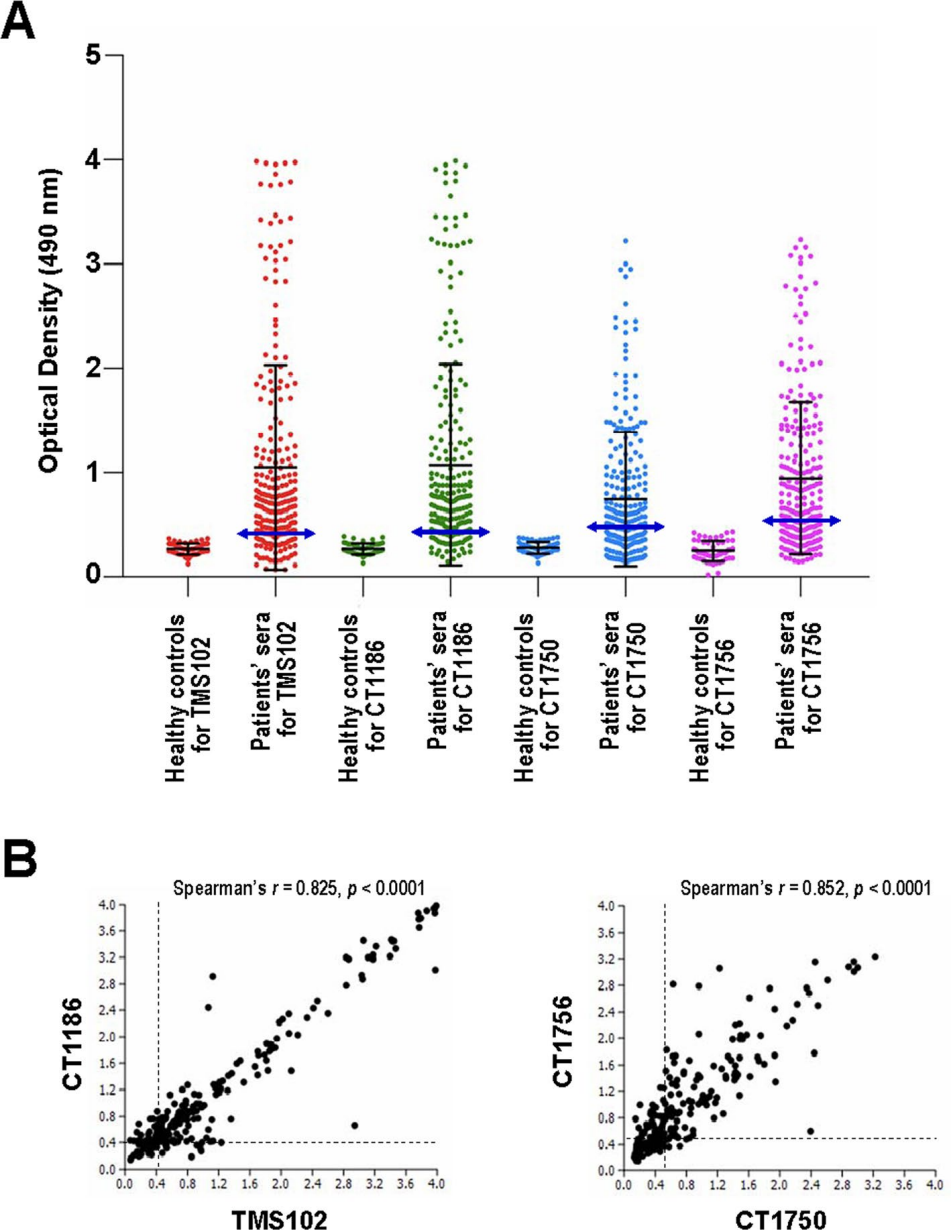
(**A**) Optical density values of total IgG antibody responses to the N- and the C-terminal PvMSP9 antigens among *P. vivax*-infected patients (n = 246) and the corresponding values in healthy control subjects (n = 20). Horizontal black lines represent means with standard errors. Double-headed arrow horizontal blue lines are cut-off values. (**B**) Pairwise comparisons of OD_490_ values for anti-PvMSP9 IgG antibodies to antigens TMS102 and CT1186, and CT1750 and CT1756. The cut-off values determined from 3 S.D. from control sera are indicated as broken lines.

Analysis of 246 serum samples from symptomatic vivax malaria patients (mean age, 30.9 years; age range, 2 to 65 years; and 204 males) from Tak and Ubon Ratchathani Provinces has shown that 216 patients (87.80%) had antibodies to one or both variants of the N-terminal antigens, i.e. TMS102 and CT1186 (Table 1, Fig. 1). Of these, 198 (80.49%) and 195 (79.27%) patients developed IgG antibodies against antigens TMS102 and CT1186, respectively. Sera from 177 patients (71.95%) were directed against both antigens. A total of 39 serum samples were reactive exclusively to either antigen TMS102 (n = 21) or CT1186 (n = 18)(Table 1). The seropositivity rates for IgG antibodies to the N-terminal antigens were not significantly different between the two provinces (χ^2^ test, *p* > 0.05). For the C-terminal antigens, the mean optical density (O.D.490) values were lower than those for the N-terminal antigens (Fig. 2A). Of these, 139 (56.50%) and 152 (61.79%) serum samples were reactive to antigens CT1750 and CT1756, respectively. Likewise, no significant difference in the seropositivity rates of antibodies to the C-terminal antigens was observed between the two provinces (χ^2^ test, *p* > 0.05) (Table 2). In total, 125 patients had IgG antibodies to both variants of the C-terminal antigens. Meanwhile, 27 patients’ sera were exclusively reactive to antigen CT1756 whereas 14 serum samples contained CT1750-specific antibodies. The seropositivity rates of antibodies to the N-terminal antigens were significantly greater than those to the C-terminal antigens (χ^2^ test, *p* < 0.0001). It is noteworthy that 154 patients (62.60%) had IgG antibodies to either or both variants of the N- and the C-terminal antigens while sera from 74 patients (30.08%) were reactive to either the N- or the C-terminal antigens (Supplemental Table S1). No antibody response to all antigens was observed in the remaining 18 patients’ sera (7.32%). Neither the seropositivity rates nor the reactivity index (RI) values showed an increasing trend toward the age groups of the patients (Supplemental Tables S2 and S3).

**Table 1:** Prevalence of IgG antibodies to the N-terminal domain of PvMSP9 among *P. vivax*-infected Thai patients.

Province	Antigen		No. reactive antigens		Exclusively reactive to antigen		
	TMS102	CT1186	≥ 1	2	TMS102	CT1186	Total
**Tak (n = 50)**							
Seropositive (%)	42 (84.00)	36 (72.00)	43 (86.00)	35 (70.00)	7 (14.00)	1 (2.00)	8 (16.00)
Seronegative (%)	8 (16.00)	14 (28.00)	7 (14.00)	15 (30.00)			
**Ubon Ratchathani (n = 196)**							
Seropositive (%)	156 (79.59)	159 (81.12)	173 (88.27)	142 (72.45)	14 (7.14)	17 (8.67)	31 (15.82)
Seronegative (%)	40 (20.41)	37 (18.88)	23 (11.73)	54 (27.55)			
**All (n = 246)**							
Seropositive (%)	198 (80.49)	195 (79.27)	216 (87.80)	177 (71.95)	21 (8.54)	18 (7.32)	39 (15.85)
Seronegative (%)	48 (19.51)	51 (20.73)	30 (12.20)	69 (28.05)

**Table 2:** Prevalence of IgG antibodies to the C-terminal domain of PvMSP9 among *P. vivax*-infected Thai patients.

Province	Antigen		No. reactive antigens		Exclusively reactive to antigen		
	CT1750	CT1756	≥ 1	2	CT1750	CT1756	Total
**Tak (n = 50)**							
Seropositive (%)	27 (54.00)	29 (58.00)	32 (64.00)	24 (48.00)	3 (6.00)	5 (10.00)	8 (16.00)
Seronegative (%)	23 (46.00)	22 (44.00)	18 (36.00)	27 (54.00)			
**Ubon Ratchathani (n = 196)**							
Seropositive (%)	112 (57.14)	123 (62.76)	134 (68.37)	101 (51.53)	11 (5.61)	22 (11.22)	33 (16.84)
Seronegative (%)	84 (42.86)	73 (37.24)	62 (31.63)	95 (48.47)			
**All (n = 246)**							
Seropositive (%)	139 (56.50)	152 (61.79)	166 (67.48)	125 (50.81)	14 (5.69)	27 (10.98)	41 (16.67)
Seronegative (%)	107 (43.50)	95 (38.62)	80 (32.52)	122 (49.59)			

### Relationship between the OD_490_ values of anti-PvMSP9 antibodies

A pairwise comparison of the OD_490_ values for total IgG antibodies to antigens TMS102 and CT1186 displayed a highly significant correlation among sera from 246 patients (Spearman’s *r* = 0.825, *p* < 0.0001). Likewise, reactivity of all serum samples to the antigens CT1750 and CT1756 has shown highly significant pairwise correlation of the OD values (Spearman’s *r* = 0.852, *p* < 0.0001) (Fig. 2B). Consistent results were obtained when seronegative samples for both variants were excluded from the analysis (Spearman’s *r* = 0.782 and 0.731, *p* < 0.0001 for the N- and the C-terminal antigens, respectively).

### Previous malaria episodes and antibody responses

To investigate whether anti-PvMSP9 antibodies could be associated with previous malaria episodes, seropositive serum samples from 174 patients with known history of previous malaria illnesses based on self-reports and/or diagnosis made by malaria clinics or local district hospitals were analyzed. Patients were categorized into 3 groups: (1) no history of previous malaria infection (n = 91), (2) a single prior malaria episode (n = 53) and (3) two or more previous episodes of symptomatic malaria (n = 30). Although the precise species of *Plasmodium* infections could not be completely obtained, vivax malaria has been most prevalent in Thailand since the past two decades and about 80% of recurrent malaria episodes were caused by the same species^20–22^. Based on this categorization, the seropositivity rates of anti-PvMSP9 antibodies to antigen TMS102 seem to increase with increasing prior malaria episodes (χ^2^ for trend, *p* = 0.001) while no significant trend was observed for antibody reactivity to other antigens (Table 3). When combined results from antibody reactivity to both variants of the N- or the C-terminal antigens were considered, a significant association between seropositivity rates and increasing prior malaria episodes was observed (χ2 for trend, *p* = 0.001 and *p* = 0.04, respectively)(Table 3). Likewise, the number of prior malaria episodes were positively associated with the levels of antibodies analyzed in terms of the reactivity index values for both variants of the N- and the C-terminal antigens (Kruskal–Wallis test, *p* = 0.049 and 0.014, respectively) (Table 4).

**Table 3:** Seropositivity rates of total IgG antibodies to PvMSP9 and previous malaria episodes.

No. prior malaria episodes	n	No. seropositives to N-terminal antigens (% responders)			No. seropositives to C-terminal antigens (% responders)		
		TMS102	CT1186	Total	CT1750	CT1756	Total
0	91	62 (68.13)	68 (74.73)	130 (71.43)	44 (48.35)	43 (47.25)	87 (47.80)
1	53	45 (84.91)	43 (81.13)	88 (83.02)	26 (49.06)	34 (64.15)	60 (56.60)
2–4	30	28 (93.33)	26 (86.67)	54 (90.00)	19 (63.33)	18 (60.00)	37 (61.67)
χ^2^ for trend		10.20	2.17	10.99	1.54	2.78	4.23
*p* value		0.001	0.14	0.001	0.22	0.10	0.04

**Table 4:** Levels and reactivity indices (RI) of anti-PvMSP9 antibodies and previous malaria episodes.

No. prior malaria episodes	n	N-terminal antigens			C-terminal antigens		
		Mean OD_490_ value (S.D.)		Mean RI for both antigens (S.D.)	Mean OD_490_ value (S.D.)		Mean RI for both antigens (S.D.)
		TMS102	CT1186		CT1750	CT1756	
0	91	1.312 (1.165)	1.311 (1.115)	3.190 (2.765)	0.910 (0.461)	1.045 (0.509)	2.035 (1.006)
1	53	1.207 (0.893)	1.240 (0.891)	2.978 (2.162)	1.017 (0.510)	1.190 (0.653)	2.301 (1.210)
2—4	30	1.508 (0.957)	1.416 (1.026)	3.571 (2.398)	1.276 (0.841)	1.487 (0.752)	2.875 (1.666)
Cut-off		0.400	0.422		0.450	0.510	
Kruskal–Wallis *H*		6.026	0.912	6.029	2.944	6.145	8.573
*p* value		0.049	0.634	0.049	0.229	0.046	0.014

### Previous malaria episodes and antibody avidity

To determine whether avidity maturation was associated with previous malaria episodes, 30 serum samples per group were randomly selected from groups I and II, and all samples from group III for analysis. It is noteworthy that the mean time from last infection among patients in group III was longer than those in group II (3.98 years vs 3.47 years). However, the mean difference was not statistical significant (Mann–Whitney *U* test, *p* > 0.05)(Supplemental Table S4). Results revealed that sera from patients in group I mainly contained low avidity anti-PvMSP9 antibodies to the N- and the C-terminal antigens while high avidity antibody was not detected. On the other hand, the majority of group II sera had intermediate avidity antibodies to these antigens. Interestingly, 22 (73.4%) and 20 (66.7%) patients in group III developed high avidity antibodies to the N- and the C-terminal antigens, respectively, while the remaining sera belonged to intermediate avidity (Table 5). There was a tendency toward developing high antibody avidity with increasing prior malaria episodes (χ^2^ for trend = 35.90, *p* < 0.0001 and 31.39, *p* < 0.0001 for the N- and C-terminal antigens, respectively). Likewise, the reactivity index values for antibodies to the N-terminal antigen have a tendency to increase with increasing malaria exposure (Kruskal Wallis *H* test, *p* = 0.036). However, such tendency was not observed for antibodies to the C-terminal antigen (Table 5).

**Table 5:** Distribution of antibody avidity and reactivity indices (RI) to the N- and the C-terminal antigens of PvMSP9 and previous malaria episodes.

No. prior malaria episodes	n	Mean age (years)	Male	N-terminal antigen			Mean RI (S.D.)	C-terminal antigen			Mean RI (S.D.)
				Antibody avidity, n (%)				Antibody avidity, n (%)			
				Low	Intermediate	High		Low	Intermediate	High	
0	30	31.0	23	20 (66.7)	10 (33.4)	0 (0)	1.77 (1.51)	19 (63.3)	11 (36.7)	0 (0)	1.41 (0.67)
1	30	27.9	28	3 (10.0)	19 (63.3)	8 (26.7)	2.32 (2.08)	3 (10.0)	20 (66.7)	7 (23.3)	1.86 (1.21)
2—4	30	28.7	23	0 (0)	8 (26.7)	22 (73.4)	2.71 (2.05)	0 (0)	10 (33.3)	20 (66.7)	1.75 (1.03)
χ^2^ for trend				34.65	0.27	35.90		32.22	0.07	31.39	
Kruskal–Wallis *H*							6.653				4.261
*p* value				< 0.0001	0.60	< 0.0001	0.036	< 0.0001	0.80	< 0.0001	0.119

### IgG subclass responses

**Figure 3:**
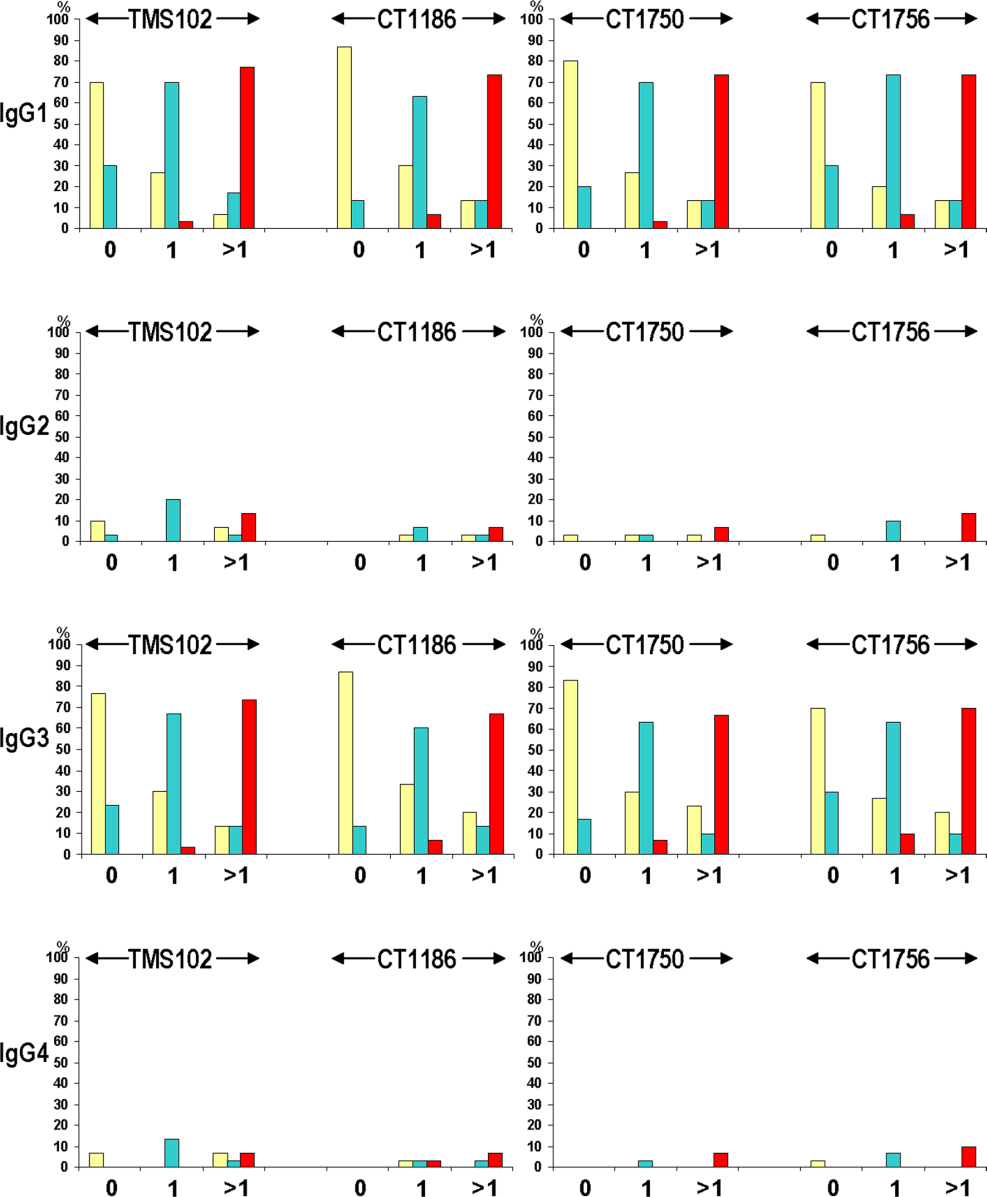
Distribution of avidity levels of IgG isotypes against PvMSP9 antigens and the number of previous malaria episodes. Vertical axis represents percentage of seropositive patients. Patients are classified as those who had prior 0, 1 and 2–4 (shown as > 1) malaria episodes. Low, intermediate and high avidity antibodies are shown in light yellow, turquoise and red bars, respectively. The percentages of high avidity IgG1 and IgG3 antibodies to all antigens display an increasing trend toward an increasing number of previous malaria episodes (χ^2^ for trend, *p* < 0.0001).

To investigate the profiles of antibody responses to the recombinant PvMSP9 antigens, IgG subclasses were determined from 90 seropositive samples used for total IgG antibody avidity assays. Results revealed that all of these seropositive sera contained both IgG1 and IgG3 antibodies against each variant of the N- and the C-terminal antigens. Of these, IgG2 antibodies to antigens TMS102 and CT1186 were detected in 56.67% and 23.33% of samples whereas 20% and 23.33% of these sera contained IgG2 antibodies to antigens CT1750 and CT1756, respectively. Meanwhile, 40%, 16.67%, 10% and 20% of these samples contained IgG4 directed against antigens TMS102, CT1186, CT1750 and CT1756, respectively (Fig. 3). The ratio of reactivity index values between cytophilic and non-cytophilic antibodies [(IgG1 + IgG3)/(IgG2 + IgG4)] seem to decrease with increasing number of previous malaria episodes, i.e. 2.439, 1.782 and 1.452 for N-terminal antigens and 2.613, 1.872 and 1.842 for C-terminal antigens. Further analysis these IgG isotypes has shown that antibodies with high avidity predominantly occurred among patients who had experienced multiple episodes of symptomatic malaria (group III). A significant association between levels of antibody avidity and the number of prior malaria exposure was observed in IgG1 and IgG3 responses to both N- and C-terminal antigens (Fig. 3 and Supplemental Table S5).

### Seroconversion among short-term follow-up sera

In total, 20 patients provided complete follow-up sera at Day 7, 14 and 21. Of these, 3 patients who were seronegative for both N-terminal antigens at Day 0 became seropositive by Day 21 while 9 of 17 seropositive patients at Day 0 had an increase in > 20% of OD_490_ values at Day 21 in comparison to the corresponding values at Day 0. Meanwhile, 5 of 8 patients who were seronegative for the C-terminal antigens at Day 0 had seroconverted at Day 21. Six of 12 patients who were seropositive for the C-terminal antigens had increasing antibody levels at Day 21. On the other hand, 3 patients who were seronegative for both C-terminal antigens remained unresponsive at Day 21 (Supplemental Table S6).

## Discussion

Several lines of evidence support that both N- and C-terminal regions of PvMSP9 were immunogenic upon natural malaria exposure. However, a considerable variation in the seropositivity rates of anti-PvMSP9 antibodies has been observed across populations and endemic areas^12,16–18,23^. Studies in Brazilian Amazon have shown a range from 16 to 80% and 50% to 73% for the seropositivity rates of antibodies to the N- and the C-terminal antigens, respectively^16–18,23^. Likewise, 45.9% and 8.7% of Papua New Guinean children had IgG antibodies to these respective antigens^12^. In this study, the seropositivity rates of anti-PvMSP9 antibodies to the N-terminal antigens were significantly higher than those to the C-terminal antigens (87.80% vs. 67.48%) in both endemic provinces of Thailand (Tables 1 and 2). Although differential seropositivity rates of anti-PvMSP9 antibodies could stem from various factors such as differences in intensity of malaria exposure^17^ and host genetic background^24^, sequence conservation in the N-terminal portion and polymorphism in the C-terminal repetitive domain of PvMSP9 could probably affect the profile of antibody responses to these domains. On the other hand, no significant difference in the seropositivity rates of antibodies to these antigens was observed between Tak and Ubon Ratchathani Provinces, suggesting that factors influencing antibody responses to PvMSP9 may not differ markedly between these endemic areas.

One of the potential hurdles for malaria vaccine development is the presence of antigenic diversity among vaccine candidates when antibodies directed against particular antigens do not react to their variants, precluding the protective efficacies. Our previous analysis of *pvmsp9* sequences among *P. vivax* isolates from diverse malaria endemic areas of Thailand has shown that the conserved N-terminal region encompassing 473 codons contained 12 amino acid substitutions^15^. Three of these substitutions occurred in antigens TMS102 and CT1186 which were located within or near the corresponding erythrocyte binding region of PfMSP9^8^ (Supplemental Fig. S1). In this study, the majority of seropositive individuals (177 of 216 patients, 81.94%) had antibodies to both antigens TMS102 and CT1186 while a highly significant correlation between the levels of antibodies to these antigens (*p* < 0.0001) may suggest the presence of common epitopes recognized by these antibodies. The remaining 39 patients (18.06%) had antibodies exclusively reactive to either one or the other N-terminal antigen, implying variant-specific antibody responses. Our previous analysis of *pvmsp9* among Thai isolates has revealed that substitution at codon 122 (D122G or N) has been under positive selection in which a consistent result was obtained by both codon-based and amino acid property-based detection methods^15^. Meanwhile, host immune responses to infections could elicit selective pressure on pathogens leading to pathogen adaptations to evade host immunity. Although the precise B cell epitopes in the N-terminal PvMSP9 remain to be elucidated, at least five T cell epitopes (pE, pH, pJ, pK and pL) have been mapped in this conserved domain but outside residues 122, 215 and 217^25^. It is likely that the 3 amino acid substitutions in antigens TMS102 and CT1186 could be the targets for variant-specific antibody recognition based on in silico linear B-cell epitope prediction using the Bepipred 2.0 server^26^ (Supplemental Fig. S2). Furthermore, peptide derived from the N-terminal part of MSP9 could bind to erythrocyte bind 3^2,3^ while interruption of this process by specific antibodies to MSP9 could exert detrimental effects for subsequent intraerythrocytic development of malaria parasites^9^. Whether variant-specific antibodies to the N-terminal PvMSP9 antigens could interfere with parasite survival would require further studies. Positive selection on microbial agents including malaria parasites by acquired immunity could be frequency-dependent selection and most of which follow inverse or negative direction. Negative frequency-dependent selection occurs when the rarer phenotype in the population or in specific environmental communities is selected for due to its greater fitness or survival advantage, resulting in adaptive polymorphisms in host-parasite coevolution^27^. Therefore, negative frequency-dependent selection is against more common phenotypes precluding fixation of alleles while maintaining genetic diversity in the population. In this study, antigen CT1186 derived from the most prevalent PvMSP9 haplotype, accounting for 50% of haplotypes circulating in Thailand, was recognized by IgG antibodies at an almost comparable rate with antibodies to antigen TMS102, a much lower prevalent haplotype (6.7%)^15^ (Table 1 and Supplemental Table S7). However, variant-specific antibodies identified herein may not exclusively recognize the epitope encompassing amino acid residue 122 but could rather contain heterogenous antibodies against one or more substituted residues in antigens TMS102 and CT1186. Therefore, the possibility of negative frequency-dependent selection on codon 122 of PvMSP9 remains inconclusive.

For the C-terminal antigens, a higher seropositivity rate of antibodies to antigen CT1756 than to antigen CT1750 was observed among patients from both Tak and Ubon Ratchathani Provinces. It is likely that more repeat units in the former antigen could contribute to more B epitopes that could be recognized by the patients’ sera (Fig. 1). It is noteworthy that almost all repeat units in domain R2 of antigen CT1750 were found in the repeat units of antigen CT1756 while the latter possessed additional distinct repeat units at the central region, i.e. A9E3A10E3A10B1E4A8D3B2D1 (Fig. 1). Meanwhile, 6 of 7 repeat units (BCBCDC) in domain R3 of antigen CT1750 were shared with the C-terminal repeat units of antigen CT1756 while extra repeat units (BCBCC) occurred in the latter antigen (Fig. 1). It is noteworthy that the shared repetitive region between these antigens was prevalent (86.54%) among *P. vivax* population in Thailand^15^ (Supplemental Table S8). Our analysis has shown that the majority (125 of 166, 75.3%) of seropositive sera were reactive to both variants of the C-terminal antigens while a significant correlation between the levels of antibodies reactive to each variant was observed (Fig. 2). Therefore, the majority of seropositive sera for the C-terminal antigens may recognize common or cross-reactive epitopes in these antigens. The higher prevalence of CT1756-specific antibodies than CT1750-specific antibodies could probably stem from specific recognition of extra repeats in the former antigen. Our previous analysis has revealed that the C-terminal region encompassing domains R2 and R3 was mainly occupied by low-complexity region (LCR), characterized by sequences with biased amino acid composition with a low diversity of residues relative to other regions of the protein^15^. It has been suggested that LCR confers phenotypic plasticity of some malarial surface proteins that may involve host-parasite interaction and could confer a role in parasite evasion of host immune responses, probably through the process of nonhomologous recombination and replication slippage^28–30^. While extensive sequence variation in domains R2 and R3 of PvMSP9 occurred among clinical isolates in Thailand^15^, it is likely that a repertoire of variant-specific antibodies could be generated upon natural *P. vivax* infections. Although the function of repetitive regions in PvMSP9 remains elusive, antibodies to repetitive sequences in malarial proteins have been proposed to be evoked by T-independent B cell activation without protective effect^31^. Consistently, naturally acquired antibodies to the C-terminal repeats of PvMSP9 did not confer protection against vivax malaria in Papua New Guinean children^12^. However, a recent linear B cell epitope mapping has revealed that antibodies to peptide E795-A808 (EAAPENAEPVHENA) or PvMSP9_(E795-A808)_ in domain R2 has been associated with extended time period since the last malaria episode whilst no such correlation was observed for antibodies to the C-terminal antigen encompassing domains R2 and R3^23^. Therefore, it is likely that the C-terminal domain of PvMSP9 contain both protective and non-protective B cell epitopes. Meanwhile, the linear B-cell epitope in the C-terminal domain of PvMSP9, designated PvMSP9_(E795-A808),_ could be a target for specific memory B cells resulting in stable levels of IgG antibodies over 6 months in the absence of re-infection^32^. It is therefore likely that the association between seropositivity rates as well as the levels of anti-PvMSP9 antibodies and the number of prior malaria episodes observed in this study could partly stem from a cumulative effect from specific memory B cells during repeated malaria infections.

Isotypic differences in IgG antibody responses to malarial proteins have important implications for vaccine development. Although it may not be generally applicable, IgG1 and IgG3 subclass responses have been associated with protection against malaria^33–35^. Both IgG1 and IgG3 are cytophilic antibodies that elicit antimalarial activities by various mechanisms such as opsonic phagocytosis, complement-mediated lysis, antibody-dependent cellular cytotoxicity and antibody-dependent respiratory burst activity by neutrophils^36–39^. Despite limited number of samples in this study, both IgG1 and IgG3 were found in all seropositive sera to either the N- or the C-terminal antigen, followed by IgG2 and IgG4. Although the association between IgG subclass responses and protection against vivax malaria was not investigated in this study, naturally acquired IgG1 antibodies to the C-terminal repeat domain of PvMSP9 seem to be associated with protection against acquiring new infection among Brazilian cohorts^17,18^. Meanwhile, IgG1 and IgG3 subclass responses to malarial antigens have been reported to be related with protein secondary structure in which the former preferentially recognized conserved regions whereas disordered regions could be targets of IgG3 response. Nevertheless, the structure of malarial protein per se may not completely dictate IgG isotype responses^40–42^. Our analysis has shown that the ratio of reactivity index values between cytophilic and non-cytophilic antibodies decreased towards an increasing number of previous malaria episodes in this study could suggest that non-cytophilic antibodies tend to developed after repeated malaria exposure. Intriguingly, the role of non-cytophilic isotype response to PvMSP9 requires further study.

Although it has been well perceived that high avidity antibodies play an important role in neutralization or inactivation of viruses and bacteria including toxins, inconsistent results have been found in malarial infections. High avidities of antibodies against *P. falciparum* apical membrane antigen 1 (PfAMA1) have been reported to confer clinical protection from subsequent infection^43^ and exert inhibitory effects on merozoite invasion of erythrocytes^44^. By contrast, antibodies against other merozoite surface proteins of *P. falciparum* seem not to be associated with the risk of acquiring malaria^45^. While the reasons behind these discrepancies remain unknown, it could be that biological function exerted by specific antibodies besides the magnitude of antibody avidity may be the crucial determinant for effective immunity against malaria. For example, high avidity antibodies to the conserved C-terminal domain of *P. falciparum* circumsporozoite protein evoked by the RTS,S/ASO1E vaccine have been associated with vaccine efficacy whilst high avidity antibodies to the tetrapeptide repeats failed to confer clinical protection against malaria in the same cohorts^46^. While the biological relevance of high avidity antibodies to PvMSP9 requires further investigation, a significant positive correlation between the magnitudes of antibody avidities to both N- and C-terminal antigens of PvMSP9 and prior malaria episodes has supported avidity maturation upon repeated exposure.

The cross-sectional prevalence of seropositivity rates of antibodies to specific malarial proteins among acutely infected patients could be potentially underestimated because seroconversion could occur during a short-term follow-up period as shown in the present study as well as in our previous report^47^. A remarkable increase in the levels of anti-malarial antibodies has been reported to occur within 1 or 2 weeks after the onset of symptoms among individuals who experienced previous malaria exposure^48–50^. However, no seroconversion was also observed in some patients in this study despite 3 weeks of follow-up period. Although a delay in seroconversion may occur beyond the follow-up period, it is possible that some patients may be immune unresponsiveness to PvMSP9. Polymorphism in the Human Leukocyte Antigens (HLA) class II loci has been found to influence humoral immune responses to PvMSP9 among people in the Brazilian Amazon. A high prevalence of seropositivity rates of anti-PvMSP9 antibodies was observed among individuals carrying HLA-DRB1*04 and HLA-DQB1*03 alleles whereas most non-responders to the C-terminal repeats had HLA-DRB1*01^24^. Meanwhile, the allele frequencies of HLA-DRB1*04, HLA-DQB1*03 and HLA-DRB1*01 among Thai population were 0.0830, 0.3288 and 0.0043, respectively^51^. Therefore, it is likely that the HLA-DRB1*01-related non-responders to the C-terminal PvMSP9 antigen may not be prevalent among Thai population.

In conclusion, the immunogenicity of both N- and C-terminal portions of PvMSP9 upon natural *P. vivax* infection has been determined among Thai patients. Most IgG antibodies to these antigens belonged to cytophilic isotype. The majority of antibodies were directed against conserved or cross-reactive epitopes while variant-specific antibodies could be detected at low frequencies. The seropositivity rates, levels and avidity of anti-PvMSP9 antibodies seem to be influenced by previous malaria episodes. As immunity against malaria requires both humoral and cellular immune responses, further investigations of both B and T cell epitopes in PvMSP9 are required for subunit vaccine development.

## Materials and methods

### Study population

A total of 246 blood samples were obtained from symptomatic *P. vivax*-infected patients who attended malaria clinics or district hospitals in Tak (GPS coordinates: 17.41878° N, 98.11889° E; n = 50) and Ubon Ratchathani Provinces (GPS coordinates: 14.58358° N, 105.42480° E; n = 196) during 2013 and 2014. Data on demography and malaria infections are shown in Supplemental Table S9. Of these, short-term follow-up blood samples at day 7, 14 and 21 were available from 20 patients from Ubon Ratchathani Province. Donors for negative control blood samples comprised 50 healthy individuals who lived in Bangkok and none had history of malaria exposure before blood sample collection. All blood samples were preserved in EDTA anticoagulant. After isolation of plasma samples, they were kept at -40 °C until use.

### Recombinant PvMSP9

Two non-overlapping domains of PvMSP9 corresponding to codons 34–367 and 641–972 were included in recombinant protein production for the N- and the C-terminal antigens, respectively (positions after the Belem sequence, GenBank accession no. AF435853). The N-terminal proteins comprised two variants derived from isolates TMS102 and CT1186 in which the former was identical with the Belem sequence and the latter contained amino acid substitutions at D122G, E215Q and R217H^15^(Fig. 1). The C-terminal peptides were derived from the PvMSP9 sequences of isolates CT1750 and CT1756 containing 253 and 347 amino acid residues, respectively. Size difference between antigens CT1750 and CT1756 stemmed from variation in the number of repeat units in the repeat domains 2 and 3^15^(Fig. 1). The 5′ fragments of *pvmsp-9* were amplified by PCR using primers PvMSP9N1F (5′-CCCC**CATATG**GCCAACCTGGTGAA CAATTACG-3′, *Nde* I site underlined) and PvMSP9N1R (5′-CCCC**CTCGAG**TTATTTG AGTTCACTAAGGTAATG-3′, *Xho* I site underlined). Amplification was performed in a total volume of 30 µL containing template DNA, 2.5 mM each deoxynucleotide triphosphate, 2.0 µL of 10X EX Taq PCR buffer, 0.3 µM each primer, and 0.5 units of Ex Taq DNA polymerase (Takara, Seta, Japan). The cycling condition for amplification contained 38 cycles of 94 °C 40 s, 55 °C 30 s and 72 °C 60 s, followed by a final extension at 72 °C 5 min. Likewise, the 3′ portion of *pvmsp9* was amplified under the same condition except that the PCR primers were replaced with PvMSP9C1F (5′-CCCC**CATATG**GTCCCAGAGAAC GTCGAAGC-3, *Nde* I site underlined) and PvMSP9C1R (5′-CCCC**CTCGAG**TTAGTCG ACGGTATTCGCGGTTTC-3′, *Xho* I site underlined). The PCR products were fractionated in 1% agarose gels and purified by QIAquick Gel Extraction Kit (Qiagen, Hilden, Germany). After digestions of the purified PCR products with *Nde*I and *Xho*I (BioLabs, Japan), they were ligated to pET19b vector and transformed into *E. coli* JM109. The recombinant plasmid DNA was verified by direct sequencing prior to transformation into *E. coli* BL21 Star (DE3) pLysS expression host (Invitrogen, Carlsbad, CA). Protein expression comprising induction with isopropyl*-β*-D thiogalactopyranoside (IPTG), solubilization by sonication, refolding by multistep dialysis and purification using His-Bind Resin (Novagen, Germany) were essentially followed the procedures previously described^47^ (Supplemental Method S1).

### Enzyme-linked immunosorbent assay (ELISA)

After the optimal antigen concentration and the dilution of the primary and secondary antibodies were determined by checkerboard cross-titrations, the plasma samples were analyzed by ELISA for the detection of specific IgG antibodies against the N- and the C-terminal antigens of PvMSP-9. The assays were performed in duplicate wells using 0.5 µg of the affinity purified antigen per well and 100 μL of 1:100 dilution of individual plasma samples. Details on the procedures were in Supplemental Method S2. The cut-off values for positive titres were determined from the arithmetic average of optical density (OD_490_) values plus 3 standard deviations (S.D.) of negative control sera from 50 healthy individuals living in Bangkok without history of malaria infection. To detect subclasses of human IgG antibodies, ELISA was performed essentially as described except that secondary antibodies were biotin-conjugated isotype-specific mouse anti-human IgG subclasses (Sigma-Aldrich, Germany) with 1:1000 dilution. The cut-off values of antibody titres for each recombinant antigen are shown in Supplemental Table S10.

### Avidity test

An ELISA for detection of total anti-IgG antibodies was carried out in duplicates, one followed the protocol essentially as described above whereas the other was assessed with a urea elution-based method as previously reported^52^. All tests were repeated twice. The avidity index was determined from the percentage of the ratio of mean OD_490_ value of urea-treated samples to that of untreated samples. The magnitudes of antibody avidity were classified as low, intermediate and high when the avidity indices were < 30%, 30%-50% and > 50%, respectively^52,53^.

### Data analysis

Quantitative data were analyzed in terms of percentage, mean and standard deviation. To compare the levels of antibodies to different antigens, the reactivity indices or RI were computed by dividing the mean OD_490_ values of tested serum samples by the cut-off values for respective antigens. Comparisons between the seropositivity rates were determined by the chi-square test. Associations between antibody responses and prior malaria episodes were performed by using the chi-square test for trend analysis and the Kruskal–Wallis test. Statistical significance was considered as *p* < 0.05. Prediction of linear B cell epitope was performed with Bepipred Linear Epitope Prediction server using the default option^26^.

### Ethical approval

This study was reviewed and approved by the Institutional Review Board in Human Research of Faculty of Medicine, Chulalongkorn University, Thailand (IRB No. 322/59 and COA No. 041/2016). Prior to blood sample collection, informed consent was obtained from all participants or from their parents or guardians. All procedures were performed in accordance to the relevant guidelines and regulations.

## Supplementary Information

Supplementary information 1.

## Data Availability

The datasets generated during and/or analyses during the current study are available from the corresponding author upon request.
