# Supplementary material for: Immunoglobulin G responses to variant forms of Plasmodium vivax merozoite surface protein 9 upon natural infection in Thailand
Source: Sci Rep. 2021 Feb 5;11:3201. doi: 10.1038/s41598-021-82928-4 (PMC7864938; doi:10.1038/s41598-021-82928-4)

Immunoglobulin G responses to variant forms of *Plasmodium vivax* merozoite surface protein 9 upon natural infection in Thailand

Sunisa Songsaigath[1,2,3], Takashi Makiuchi[1], Chaturong Putaporntip[2], Urassaya Pattanawong[2], Napaporn Kuamsab[2], Hiroshi Tachibana[1], Somchai Jongwutiwes[2]

[1]Department of Infectious Diseases, Tokai University School of Medicine, Isehara, Kanagawa, Japan; [2]Molecular Biology of Malaria and Opportunistic Parasites Research Unit, Department of Parasitology, Faculty of Medicine, Chulalongkorn University, Bangkok, Thailand. [3]Inter-Department Program of Biomedical Sciences, Faculty of Graduate School, Chulalongkorn University, Bangkok, Thailand

SUPPLEMENTARY INFORMATION

Contents

Method S1. Expression, purification and analysis of recombinant PvMSP9 proteins…………......3

Method S2. Enzyme-linked immunosorbent assay……………………………………..……5

Table S1. Profile of IgG antibodies to the N- and the C-terminal PvMSP9 antigens among 246 *P. vivax*-infected patients……………………………………….………………..6

Table S2. Seropositivity rates of antibodies to PvMSP9 antigens across age groups…………..7

Table S3. Mean reactivity indices (RI) of antibodies to PvMSP9 antigens across age groups....8

Table S4. Time from last infection and previous malaria episodes…………….……………….9

Table S5. Avidity levels of IgG isotypes to the N- and the C-terminal antigens of PvMSP9 and previous malaria episodes…………………...………………………………….10

Table S6. Changes in serostatus and levels of anti-PvMSP9 antibodies among 20 vivax malaria patients………………………………...…………………………………11

Table S7. Prevalence of PvMSP9 alleles based on amino acid residues 122, 215 and 217…...12

Table S8. Prevalence of the C-terminal PvMSP9 antigens CT1750 (R2-III and R3-XXXIX)

and CT1756 (R2-VI and R3-IV)………………………….....…………………...13

Table S9. Demographic data of *P. vivax*-infected patients………………….....……………...15

Table S10. Optical density and cut-off values of total IgG and IgG subclasses for N- and C-terminal PvMSP9 antigens among 20 healthy control sera……………….....…….16

Figure S1. Conserved domain I of PvMSP9 (Belem strain) and its corresponding sequences of *P. cynomolgi* (PcyMSP1), *P. knowlesi* (PkMSP9) and *P. falciparum* (PfMSP9)…..17

Figure S2. Plot of linear B cell epitope probability across the PvMSP9 sequence of isolate TMS102…………………………………… …………………………...…….18

Method S1. Expression, purification and analysis of recombinant PvMSP9 proteins.

1. Expression of the N- and the C-terminal PvMSP9 constructs

The recombinant proteins were expressed as described previously [47]. In brief, protein expression was induced with 1 mM of isopropyl-*β*-D thiogalactopyranoside (IPTG) at 30°C for 6 h under constant agitation. For recombinant proteins produced in inclusion bodies, the cell pellets were re-suspended in 20 μL of 1M Tris-HCl pH 7.5, 250 mM EDTA, 1% Triton X-100 and 250 mM phenylmethylsulfonyl fluoride (PMSF), vortexed and sonicated on ice. After adding lysozyme, the lysates were incubated for 60 min at 30°C under gentle shaking, sonicated on ice and washed for 5 times with buffer containing 1M Tris-HCl pH 7.5, 250 mM EDTA and 1% Triton X-100. The cell pellets were dissolved in solubilization buffer containing 500 mM 3-(cyclohexyamino)-1 propanesulfonic acid (CAPS) pH 11, 1, 1M dithiothreitol (DTT) and 5% N-lauroylsarcosine.

2. Purification of recombinant proteins

Purification of recombinant PvMSP9 proteins essentially followed the procedures reported by Kosuwin and colleagues [47]. After cell debris was removed from the protein suspension by centrifugation, refolding of recombinant PvMSP-9 proteins was carried out by multistep dialysis of solubilized inclusion bodies. An initial step was done in 2 liters of dialysis buffer (1M Tris–HCl pH 7.4) with 0.1mM DTT for 3 h at 4°C, followed by an overnight dialysis at 4°C with fresh buffer. The solubilized protein was then dialyzed in buffer without DTT for 3 h and repeated twice. Final dialysis was performed in redox refolding buffer containing 0.2 mM oxidized glutathione and 1 mM reduced glutathione at 4°C overnight. The purified recombinant PvMSP-9 proteins were aliquoted and kept at -80°C until use. Meanwhile, purification of soluble proteins was done by affinity chromatography using His-Bind Resin (Novagen, Germany) according to the manufacturer's recommendation. In brief, the cell pellets were harvested by centrifugation at 6,000 g for 30 min at 4°C, allowed to drain dry completely and resuspended in binding buffer containing 20 mM Tris-HCl pH 7.5, 5 mM imidazole, 500 mM NaCl and 250 mM

PMSF. After vortexing and sonicating on ice, lysozyme solution (20 mg/ml) was added, incubated at 30°C 60 min and sonicated again. After centrifugation at 6000 g for 30 min at 4°C, the supernatant was passed through a filter with pore size of 0.45 μm and kept at room temperature for 30 min before further purification with His-Bind Resin column following the manufacturer's recommendation and kept at -80 °C until use. Protein concentration was estimated using *DC* Protein Assay (Bio-Rad, Japan).

3. Analysis of recombinant PvMSP9 proteins

Recombinant proteins were analyzed by using 12% sodium dodecyl sulfate-polyacrylamide gel electrophoresis (SDS-PAGE) under reducing (with β-mercaptoethanol) and non-reducing (without β-mercaptoethanol) conditions with a standard molecular weight maker. Electrophoresis was performed at a constant voltage (100V). The gel was stained with Coomassie Brilliant Blue solution for 30 minutes at room temperature and detained with solution containing 10% glacial acetic and 30% methanol until the protein bands could be clearly visible.

Method S2 Enzyme-linked immunosorbent assay (ELISA).

The procedure for ELISA was performed as described previously by Kosuwin and colleages [47]. In brief, PolySorp immunoplates (Nunc, Wiesbaden, Germany) were coated in duplicate wells with 0.5 μg per well of the affinity purified PvMSP-9 protein in a 0.06 M carbonate-bicarbonate buffer containing 15mM Na_2CO_3, 35mM $NaHCO_3$, 0.2 M NaCl pH 9.6, and incubated at 4°C overnight. After washing with phosphate buffer saline (PBS) buffer containing 0.05% Tween 20 (PBS/T), the microplates were blocked with of 3% skim milk in PBS at room temperature for 1 h, washed with PBS buffer and incubated with 100 μl of individual plasma samples in duplicate wells at 1:100 dilutions at room temperature for 1 h. After washing with PBS/T, the plates were incubated with horseradish peroxidase-conjugated goat anti-human IgG diluted to 1:1000 with 3% skimmed milk for 1 h at room temperature and washed again with PBS/T. The presence of bound IgG antibodies was detected by using o-phenylenediamine dihydrochloride-hydrogen peroxide (Sigma-Aldrich, Germany) and the reaction was terminated with 2 N sulphuric acid. The reaction was analyzed by measurement of absorbance at 490 nm using a microplate reader (Biotech, Winooski, VT, USA).

Table S1. Profile of IgG antibodies to the N- and the C-terminal PvMSP9 antigens among 246 *P. vivax*-infected patients.

Category	N-terminal antigen		C-terminal antigen		n	%
	TMS102	CT1186	CT1750	CT1756		
I	+	+	+	+	102	41.46
II	+	+	+	-	7	2.84
III	+	+	-	+	17	6.91
IV	+	-	+	+	12	4.88
V	-	+	+	+	4	1.63
VI	+	-	+	-	1	0.41
VII	+	-	-	+	4	1.63
VIII	-	+	+	-	2	0.81
IX	-	+	-	+	5	2.03
X	+	+	-	-	51	20.73
XI	+	-	-	-	4	1.63
XII	-	+	-	-	7	2.84
XIII	-	-	+	+	7	2.84
XIV	-	-	+	-	4	1.63
XV	-	-	-	+	1	0.41
XVI	-	-	-	-	18	7.32

+, positive response; -, negative response.

Categories I – IX represent positive antibodies to one or both variants of the N- and the C-terminal antigens (n = 154).

Categories X – XV display positive antibodies to either the N- or the C-terminal antigens (n = 74).

Category XVI denotes no antibody response to both N- and C-terminal antigens (n = 18).

Table S2. Seropositivity rates of antibodies to PvMSP9 antigens across age groups

**Age (yr)**	**n**	**Seropositivity rates (%) to antigens**			
		**TMS102**	**CT1186**	**CT1750**	**CT1756**
0-10	17	64.71	76.47	47.06	47.06
11-20	37	81.08	83.78	48.65	54.05
21-30	62	82.26	79.03	61.29	67.74
31-40	51	88.24	80.39	58.82	66.67
41-50	62	75.81	79.03	58.06	62.90
>50	17	82.35	70.59	52.94	52.94
**Total**	**246**				
**χ2 for trend**		2.307	1.116	3.372	3.148
***p* value**		0.129	0.291	0.066	0.076

Table S3. Mean reactivity indices (RI) of antibodies to PvMSP9 antigens across age groups

Age (yr)	n	Mean RI for antigens					
		TMS102	CT1186	Both N-antigens	CT1750	CT1756	Both C-antigens
0-10	17	2.305	2.296	2.301	1.424	1.694	1.559
11-20	37	2.965	2.735	2.85	1.727	1.71	1.718
21-30	62	2.665	2.479	2.572	1.662	1.851	1.757
31-40	51	2.598	2.464	2.531	1.518	1.992	1.755
41-50	62	2.553	2.69	2.621	1.778	1.922	1.85
>50	17	2.448	2.344	2.396	1.651	1.678	1.665
**Total**	**246**						
**$\chi 2$ for trend**		0.943	1.918	1.379	2.675	2.839	2.770
***p* value**		0.331	0.166	0.240	0.102	0.092	0.096

Table S4. Time from last infection and previous malaria episodes

	No. previous malaria episodes	
	Single	2 - 4
n	30	30
Time from last infection (year)		
Range	2 - 7	2 – 8
Mean ± S.D.	3.47 ± 1.41	3.98 ± 1.60
Median	3	4
Difference between mean	0.51	
Mann-Whitney *U* test	363	
Mean rank	13.8	16.7
z	-1.304	
p value	0.192	

Table S5. Avidity levels of IgG1 and IgG3 isotypes to the N- and the C-terminal antigens of PvMSP9 and previous malaria episodes.*

Antigen	IgG isotype	Avidity	No. prior malaria episodes and no. of responders			χ^2 for trend	*p* value
			0 (n =30)	1 (n = 30)	2-4 (n= 30)		
TMS102	IgG1	Low	21	8	2	26.35	<0.0001
		Intermediate	9	21	5	1.11	0.29
		High	0	1	23	44.58	<0.0001
CT1186	IgG1	Low	26	9	4	32.49	<0.0001
		Intermediate	4	19	4	0	1.00
		High	0	2	22	40.79	<0.0001
CT1750	IgG1	Low	24	8	4	27.47	<0.0001
		Intermediate	6	21	4	0.29	0.59
		High	0	1	22	41.93	<0.0001
CT1756	IgG1	Low	21	6	4	21.09	<0.0001
		Intermediate	9	22	4	1.73	0.19
		High	0	2	22	40.79	<0.0001
TMS102	IgG3	Low	23	9	4	24.79	<0.0001
		Intermediate	7	20	4	0.66	0.42
		High	0	1	22	41.93	<0.0001
CT1186	IgG3	Low	26	10	6	26.49	<0.0001
		Intermediate	4	18	4	0	1.00
		High	0	2	20	35.69	<0.0001
CT1750	IgG3	Low	25	9	7	21.53	<0.0001
		Intermediate	5	19	3	0.31	0.57
		High	0	2	20	35.69	<0.0001
CT1756	IgG3	Low	21	8	6	15.60	<0.0001
		Intermediate	9	19	3	2.63	0.11
		High	0	3	21	37.17	<0.0001

* Note: The number of responders for IgG2 and IgG4 was too small for analysis.

Table S6. Changes in serostatus and levels of anti-PvMSP9 antibodies among 20 vivax malaria patients.

Status	PvMSP9 antigens	
	N-terminal domain (%)	C-terminal domain (%)
Seronegative at Day 0	3	8
Seroconversion at Day 7	1 (33.3)	3 (37.5)
Day 14	1 (33.3)	1 (12.5)
Day 21	1 (33.3)	1 (12.5)
No seroconversion (Day 21)	0 (0)	3 (37.5)
Seropositive at Day 0	17	12
>20% increased antibody titre at Day 21 relative to Day 0	9 (52.9)	6 (50.0)
No change in antibody titre at Day 21	8 (47.1)	6 (50.0)

Table S7. Prevalence of PvMSP9 alleles based on amino acid residues 122, 215 and 217.*

Allele	Amino acid at codons			n	%
	122	215	217		
**I**	**G**	**Q**	**H**	**52**	**50.00**
II	D	Q	H	14	13.46
III	D	E	H	13	12.50
IV	G	E	R	9	8.65
V	G	E	H	7	6.73
**VI**	**D**	**E**	**R**	**7**	**6.73**
VII	N	E	R	2	1.92
Total				104	100

* Data from [15]. Sequences CT1186 and TMS102 belonged to alleles I and VI, respectively.

Table S8. Prevalence of the C-terminal PvMSP9 antigens CT1750 (R2-III and R3-XXXIX) and CT1756 (R2-VI and R3-XIV).*

R2 Allele	Amino acid sequence#	Total(%)
R1-I	A1 E1 A2 E2 A3 A1 E5 A9 E3 A10 B1 E4 A8 D3 B2 D1 A2 D2 A4 A6 C1 A5 C1 A5 C1 A5 C1 A5 C1 A5 C2	0
R2-II	A1 E1 A2 E2 A3 A1 E5 A9 E3 A10 B1 E4 A8 D3 B2 D1 A2 D2 A4 A5 C2 A6 C2 A6 C1 A6 C2 A5 C2 A5 C2	2 (1.92)
**R2-III**	**A1 E1 A2 E2 A3 A1 E5 A9 E3 A10 E3 A10 B1 E4 A8 D3 B2 D1 A2 D2 A4 A5 C1 A5 C2 A6 C1 A5 C3 C2**	**1 (0.96)**
R2-IV	A1 E1 A2 E2 A3 A1 E5 A9 E3 A10 B1 E4 A8 D3 B2 D1 A2 D2 A4 A5 C1 A5 C2 A6 C1 A5 C2 A5 C3 C2	1 (0.96)
R2-V	A1 E1 A2 E2 A3 A1 E5 A9 E3 A10 B1 E4 A8 D3 B2 D1 A2 D2 A4 A5 C1 A5 C2 A5 C3 C3 C3 C3 C2	2 (1.92)
R2-VI	A1 E1 A9 E2 A3 A1 E5 A9 E3 A10 B1 E4 A8 D3 B2 D1 A2 D2 A4 A5 C1 A5 C2 A6 C1 A5 C3 C3 C2	1 (0.96)
R2-VII	A1 E1 A2 E2 A3 A1 E5 A9 E3 A10 B1 E4 A8 D3 B2 D1 A2 D2 A4 A5 C1 A5 C2 A6 C1 A5 C3 C3 C2	2 (1.92)
R2-VIII	A1 E1 A2 E2 A3 A1 E5 A9 E3 A10 B1 E4 A8 D3 B2 D1 A2 D2 A4 A5 C1 A4 A6 C1 A5 C1 A5 C3 C2	1 (0.96)
R2-IX	A1 E1 A2 E2 A3 A1 E5 A9 E3 A10 B1 E4 A8 D3 B2 D1 A2 D2 A4 A5 C1 A5 C2 A6 C1 A5 C3 C2	11 (10.58)
R2-X	A1 E1 A2 E2 A3 A1 E5 A9 E3 A10 B1 E4 A8 D3 B2 D1 A2 D2 A4 A5 C1 A5 C1 A6 C1 A5 C3 C2	22 (21.15)
R2-XI	A1 E1 A2 E2 A3 A1 E5 A9 E3 A10 B1 E4 A8 D3 B2 D1 A2 D2 A5 C1 A5 C1 A5 C2 A6 C1 A5 C2	1 (0.96)
R2-XII	A1 E1 A2 E2 A3 A1 E5 A9 E3 A10 B1 E4 A8 D3 B2 D1 A2 D2 A4 A5 C1 A5 C1 A6 C1 A5 C2	2 (1.92)
R2-XIII	A1 E1 A2 E2 A3 A1 E5 A9 E3 A10 B1 E4 A8 D3 B2 D1 A2 D2 A4 A5 C1 A5 C1 A6 C1 A6 C2	0
R2-XIV	A1 E1 A2 E2 A3 A1 E5 A9 E3 A10 B1 E4 A8 D3 B2 D1 A2 D2 A4 A5 C1 A5 C2 A6 C1 A5 C2	5 (4.81)
R2-XV	A1 E1 A2 E2 A3 A1 E5 A9 E3 A10 B1 E4 A8 D3 B2 D1 A2 D2 A4 A5 C1 A5 C2 A5 C2 A6 C2	1 (0.96)
R2-XVI	A1 E1 A2 E2 A3 A1 E5 A9 E3 A10 B1 E4 A8 D3 B2 D1 A2 D2 A4 A5 C1 A6 C1 A5 C3 C3 C2	1 (0.96)
R2-XVII	A1 E1 A2 E2 A3 A1 E1 A9 E3 A10 B1 E4 A8 D3 B2 D1 A2 D2 A4 A5 C2 A6 C2 A6 C3 C3 C2	1 (0.96)
R2-XVIII	A1 E1 A2 E2 A3 A1 E5 A9 E3 A10 B1 E4 A8 D3 B2 D1 A2 D2 A4 A5 C1 A7 C1 A5 C1 A5 C2	1 (0.96)
R2-XIX	A1 E1 A2 E2 A3 A1 E5 A9 E3 A10 B1 E4 A8 D3 B2 D1 A2 D2 A5 C1 A5 C2 A6 C1 A5 C2	1 (0.96)
R2-XX	A1 E1 A2 E2 A3 A1 E5 A9 E3 A10 B1 E4 A8 D3 B2 D1 A2 D2 A4 A5 C3 C3 C1 A5 C3 C2	8 (7.69)
R2-XXI	A1 E1 A2 E2 A3 A1 E5 A9 E3 A10 B1 E2 A3 A1 E5 A2 D2 A4 A5 C2 A6 C1 A5 C3 C2	3 (2.89)
R2-XXII	A1 E1 A2 E2 A3 A1 E5 A2 E3 A10 B1 E4 A8 D3 B2 D1 A2 D2 A4 A4 A4 A5 C1 A5 C2	0
R2-XXIII	A1 E1 A2 E2 A3 A1 E5 A9 E3 A10 B1 E4 A8 D3 B2 D1 A2 D2 A4 A4 A5 C2 A4 A5 C2	1 (0.96)
R2-XXIV	A1 E1 A2 E2 A3 A1 E5 A9 E3 A10 B1 E4 A8 D3 B2 D1 A2 D2 A4 A5 C3 C3 C2 A6 C2	1 (0.96)
R2-XXV	A1 E1 A2 E2 A3 A1 E5 A9 E3 A10 B1 E4 A8 D3 B2 D1 A2 D2 A4 A5 C2 A6 C1 A5 C2	7 (6.73)
R2-XXVI	A1 E1 A2 E2 A3 A1 E5 A9 E3 A10 B1 E4 A8 D3 B2 D1 A2 D2 A4 A6 C1 A5 C1 A5 C3	0
R2-XXVII	A1 E1 A2 E2 A3 A1 E5 A9 E3 A10 B1 E4 A8 D3 B2 D1 B2 D1 A2 D2 A4 A5 C1 A5 C2	5 (4.81)
R2-XXVIII	A1 E1 A2 E2 A3 A1 E5 A9 E3 A10 B1 E4 A8 D3 B2 D1 B2 D1 A2 D2 A5 C1 A5 C2	0
R2-XXIX	A1 E1 A2 E2 A3 A1 E5 A9 E3 A10 B1 E4 A8 D3 B2 D1 A2 D2 A4 A5 C1 A5 C3 C2	1 (0.96)
R2-XXX	A1 E1 A2 E2 A3 A1 E5 A9 E3 A10 B1 E4 A8 D3 B2 D1 A2 D2 A4 A5 C3 C1 A5 C2	1 (0.96)
R2-XXXI	A1 E5 A9 E3 A10 B1 E4 A8 D3 B2 D1 A2 D2 A4 A5 C2 A6 C1 A5 C4 C1 A5 C3 C2	0
R2-XXXII	A1 E1 A2 E2 A3 A1 E5 A9 E3 A10 B1 E4 A8 D3 B2 D1 A2 D2 A4 A5 C1 A5 C2	4 (3.85)
R2-XXXIII	A1 E1 A2 E2 A3 A1 E5 A9 E3 A10 B1 E4 A8 D3 B2 D1 A2 D2 A4 A5 C1 A6 C2	2 (1.92)
R2-XXXIV	A1 E1 A3 A1 E5 A9 E3 A10 B1 E4 A8 D3 B2 D1 B2 D1 A2 D2 A4 A5 C1 A5 C2	1 (0.96)
R2-XXXV	A1 E1 A2 E2 A3 A1 E5 A9 E3 A10 B1 E4 A8 D3 B2 D1 A2 D2 A4 A4 A4 A5 C2	2 (1.92)
R2-XXXVI	A1 E1 A2 E2 A3 A1 E5 A9 E3 A10 B1 E2 A3 D3 B2 D1 A2 D2 A4 A5 C1 A5 C2	10 (9.62)
R2-XXXVII	A1 E1 A2 E2 A3 A1 E5 A9 E3 A10 B1 E4 A8 D3 B2 D1 A2 D2 A4 A5 C3 C2	0
R2-XXXVIII	A1 E1 A2 E2 A3 A1 E5 A9 E3 A10 B1 E4 A8 D3 B2 D1 A2 D2 A4 A5 C1	1 (0.96)
**R2-XXXIX**	**A1 E1 A2 E2 A3 A1 E5 A2 D2 A4 A5 C1 A5 C2 A6 C1 A5 C3 C3 C3 C2**	**1 (0.96)**
R2-XL	A1 E5 A9 E3 A10 B1 E4 A8 D3 B2 D1 A2 D2 A4 A5 C1 A3	0

#A1, PENVEAVTE; A2, PENAEVA; A3, PENAEAV; A4, PENAEAA; A5, PENAEPV; A6, PENADPV; A7, PENANPV; A8, PENTETV; A9, PENTEVA; A10, PEDAEAA; B1, PEHTEVT; B2, PEHTEVA; C1, HENAEAA; C2, HENAEAV; C3, HENAEPV; C4, HENADPV; D1, TEVVEVV; D2, TEVVEAV; D3, PEVVEAV; E1,TEEVVEAA; E2, TEEVVEAT; E3, TEEVVEAV; E4, TEEVVETA and E5, TEEVVEVV. Thai isolates previously reported are in paraentheses. Representative sequences for haplotypes absence from this study are R2-I, Belem (GenBank accession no. AF435853); R2-XIII, India VII;R2-XXII (AFBK01002032.1), Brazil I;R2-XXVI (AFMK01001444.1), Salvador I;R2-XXVIII (PVX_124060), PvThai2A (KC935389);R2-XXXI, PvThai40B (KC935391);R2-XXXVII, PvThai67E (KC935394) and R2-XL, Mauritania (AFNI01001312.1). Alleles for CT1750 and CT1756 are in bold.

R3 Allele	Amino acid sequences##	Total (%)
R3-I	A B C B C B C D C D C B C D C	1 (0.96)
R3-II	A B C D C D C D C B C D C D C	1 (0.96)
R3-III	B C D C D C D C B C D C D C	10 (9.62)
R3-IV	B C B C B C B C D C D C	2 (1.92)
R3-V	A B C B C B C B C D C	1 (0.96)
**R3-VI**	**B C B C C B C B C D C**	**1 (0.96)**
R3-VII	B C B C B C B C D C	28 (26.92)
R3-VIII	A B C B C B C D C	26 (25.00)
R3-IX	B C B C C B C D C	0 (0)
R3-X	A B C B C D C D C	4 (3.85)
R3-XI	A A B C B C D C	1 (0.96)
R3-XII	B C B C B C D C	1 (0.96)
R3-XIII	B B C A C D C	1 (0.96)
**R3-XIV**	**A B C B C D C**	**22 (21.15)**
R3-XV	A B C B D C	1 (0.96)
R3-XVI	B C B C D C	3 (2.88)
R3-XVII	A B C D C	1 (0.96)

##A, AVPESVE; B, AVPESAE; C, AVTEAVE; D, AVPENTE. Haplotype R3-IX from GenBank accession no. KC935392. The N- and C-termini of domain 3 are VE and AAPE, respectively. Alleles for CT1750 and CT1756 are in bold.

*Data from [15].

Table S9. Demographic data of *P. vivax*-infected patients

**Province**		**Tak (n = 50)**	**Ubon Ratchathani (n = 196)**	**Total (n = 246)**
**Gender**	Male	30	174	204
	Female	20	22	42
**Age (years)**	≤ 10	14	3	17
	11-20	12	25	37
	21-30	13	49	62
	31-40	4	47	51
	41-50	1	48	49
	> 50	6	24	30
**Ethnicity**	Thai	24	195	219
	Myanmar	4	0	4
	Karen	9	0	9
	Laos	0	1	1
	Cambodia	13	0	13
**Duration of fever prior to diagnosis (days)**	1	5	16	21
	2	10	22	32
	3	19	28	47
	4	5	10	15
	5	3	3	6
	6	1	0	1
	7	3	3	6
	> 7	1	0	1
	No data	3	114	117
**No. previous malaria episodes**	0	9	82	91
	1	8	45	53
	2	5	8	13
	3	9	1	10
	4	4	3	7
	No data	15	57	72

Table S10. Optical density and cut-off values of total IgG and IgG subclasses for N- and C-terminal PvMSP9 antigens among 20 healthy control sera.

Antigen	Antibody	O.D. (490 nm)				
		Minimum	Maximum	Mean	S.D.	Cut-off
TMS102	Total IgG	0.123	0.366	0.247	0.051	0.400
	IgG1	0.053	0.164	0.091	0.026	0.169
	IgG2	0.027	0.136	0.064	0.031	0.157
	IgG3	0.014	0.114	0.063	0.027	0.144
	IgG4	0.017	0.167	0.068	0.033	0.167
CT1186	Total IgG	0.130	0.387	0.266	0.052	0.422
	IgG1	0.034	0.146	0.062	0.024	0.134
	IgG2	0.024	0.178	0.058	0.032	0.154
	IgG3	0.034	0.113	0.069	0.022	0.135
	IgG4	0.012	0.112	0.066	0.027	0.147
CT1750	Total IgG	0.131	0.378	0.279	0.057	0.450
	IgG1	0.036	0.104	0.052	0.019	0.109
	IgG2	0.038	0.165	0.075	0.030	0.165
	IgG3	0.024	0.167	0.067	0.035	0.172
	IgG4	0.035	0.127	0.057	0.024	0.129
CT1756	Total IgG	0.019	0.431	0.242	0.090	0.512
	IgG1	0.034	0.124	0.061	0.026	0.139
	IgG2	0.024	0.165	0.087	0.043	0.216
	IgG3	0.036	0.198	0.075	0.040	0.195
	IgG4	0.034	0.147	0.070	0.034	0.172

Figure S1. Conserved domain I of PvMSP9 (Belem strain) and its corresponding sequences of *P. cynomolgi* (PcyMSP9), *P. knowlesi* (PkMSP9) and *P. falciparum* (PfMSP9)(GenBank accession nos AF435853, , KC935411, AF435855 and LN999947, respectively). Erythrocyte-binding regions in PfMSP9 [8] are shown in red. Amino acid substitutions among Thai *P. vivax* isolates are shown underneath the Belem sequence whose identical residues are indicated by dots [15]. The region for the N-terminal antigen is marked with asterisks above the alignment. Amino acid substitutions in antigens TMS102 and CT1186 are shaded.

```
                                                   ← N-terminal antigen ****************************************************
PvMSP9      MRVNIIIFSF VVFLVKGHVA KSDDKKSFSK LFDANLVNNY EKLFKVIKCQ YC-----LTQ KKGAQNEKCK QIMKDCKKLL KDGEYISTLK DLMNDLKVGK  [ 95]
  variants  .......... L......... ......N... .......... .......... ..-----... .......... .......... .......... ..........
PcyMSP9     MRASIIILSF VVFLVRGHIV KADDKKSFSK LFDANLVNNY EKLFKVIKCQ YC-----LTQ KKGAQNEKCK KIMKECEHLL KDGEYITTLK DLMNDLKVGN  [ 95]
PkMSP9      MRVSIIIFSF VVFLVKGHIA KSDDKKNFSK LFDANLVNNY EKLFKVIKCQ YC-----LTQ KKGAQNEKCK KIMKECKHLL KDGEYMTMLK DLMSDLKVGK  [ 95]
PfMSP9      MNMKIVLFSL LLFVIRWNII SC-NKNDKNQ GVDMNVLNNY ENLFKFVKCE YCNEHTYVKG KKAPSDPQCA DIKEECKELL KEKQYTDSVT YLMDGFKSAN  [ 99]

            ****************************************************************************************************************
PvMSP9      NEHVYGP--N TNELKKIINF LELHKQEIDM LKDMIDTIKD NKAILLINGG NNPVVHKLNK KMKSLKKSVE YIQKSQDVIT EQLSK----- ----------  [178]
  variants  .......--. .......... ........G. .......... .......... .......... .......... .......... .....----- ----------
PcyMSP9     NDHVYGP--N TKELKKIINF LELHMQEIGK LKDMIDTIKD NKAILLVNGG NNPVIHKLNK KMKSLKNNVE YIQKSQDIIT EQISK----- ----------  [178]
PkMSP9      NEHVYGP--N TEELKKIINF LELHKQEIKK IKNMIDTIKN NKAILLINGG DNAMVHKLNK KMKSLKNGVE YIQKSQEAIT KHLSE----- ----------  [178]
PfMSP9      NSANNGKKNN AEEMKNLVNF LQSHKKLIKA LKKNIESIQN KKHLIYKNKS YNPLLLSCVK KMNMLKENVD YIQKNQNLFK ELMNQKATYS FVNTKKKIIS  [199]

            ****************************************************************************************************************
PvMSP9      ---------- -------HED GVNHVLSETF HLKGSTNKDG NELIQLGNVT ENDEERN--- ---------- ---------- -YELGGLGVD EFVKNGIKDL  [237]
  variants  ---------- -------... .......... .........D .......... ...Q.H.--- ---------- ---------- -......... ..........
PcyMSP9     ---------- -------NDD EENNVLPDMF HLNATINKDD NELIQLGNIA DNDEEHK--- ---------- ---------- -FELGGVDID EFVKNSMKDL  [237]
PkMSP9      ---------- -------NDD EVNNILPDMF HLKGTTNKNG NELIQLGNVT DTDGENN--- ---------- ---------- -YELGNLGVE DVIKNSMKGL  [237]
PfMSP9      LKSQGHKKET SQNQNENNDN QKYQEVNDED DVNDEEDTND DEDTNDEEDT NDDEDTNDDE DTNDEEDTND EEDHENNNAT AYELGIVPVN DVLNVNMKNM  [299]

            ****************************************************************************************************************
PvMSP9      FKDGEGFMDV VKTTLIQEGG GIGGELVSLI EKGKEIGEKI VDIEGMITNK HSTGNTDGTT IEKLDHFKTE LSSYEFLLIS LKGIVLSKLK DILLRVLYKT  [337]
  variants  .......... .......... .......... .......... .......... ..I....... .......... .......... .......... .....L....
PcyMSP9     FKDGEGFMDA VKTTLIQEGG GIGGELVSLI EKGKEIGEKI VDIEGMITNK NNPANKDGTT IEKLDHFKTE LSSYEFLIIS LKGIVLSKLK DILLRLLYKA  [337]
PkMSP9      FKDGEGLMDV VKNTLMQEGG GIGGELVSLI EKGKEIGQKI VDIEGMITNK DGTANADGTT IEKLDHFKTE LSSYEFLITS LKGIVLGKLK DILLRLLYKA  [337]
PfMSP9      IT-GNNFMDV VKNTLAQSGG LGSNDLINFL NQGKEIGENL LNITKM--NL GDKNNLESFP LDELNMLKDN LINYEFILDN LKTSVLNKLK DLLLRLLYKA  [396]

            *********** N-terminal antigen →
PvMSP9      YINYRTKKAK EFGEPAPTVV SEEHYLSELK KGVLELGMKI LFNKLRNLLT IVKKKMFPKK FGGKKGKTEK EDTQADPNAA TPETAELSSQ MLRGSVPAED  [437]
  variants  .......... .......... .......... .......... .......... .........M ....ED.... .N........ .......... ..........
PcyMSP9     YISYRTKKAI EFEEPPPTEV SEEHYLSELK KGVLELGMKI LFNKLRNLLT IVKKKLFPKK AGSKGGKTEK EQKKGDSKTA TPETAELSSQ MLRGSVPAED  [437]
PkMSP9      YISYKTKKAK EFGEPVPSEV PEEEYLNELK KGGLELGIKI LFNKLRYLLI VIKKKLFPKK FGNKEDKTKK GENKALPKSV TLESAKLNSN MLRGSVCEED  [437]
PfMSP9      YVSYKKRKAQ EKGLPEPT-V TNEEYVEELK KGILDMGIKL LFSKVKSLLK KLKNKIFPKK ---------K EDNQAVDTKS MEEPKVKAQP ALRGVEPTED  [486]


PvMSP9      VSLMASIDSM IDEIDFYEKE IYYNPHTGAV AQVHGM [537]
  variants  .......... .......... .......... ......
PcyMSP9     FSLMASIDSM IEEIDFYEKE IYHNPHTQEV MDGGHK [537]
PkMSP9      VSLMTSIDNM IEEIDFYEKE IYKGSHSGGV IKGMDY [537]
PfMSP9      SNIMNSINNV MDEIDFFEKE LIENNNTPNV VPPTQS [545]
```

Figure S2. Plot of linear B cell epitope probability across the PvMSP9 sequence of isolate TMS102. Broken red line indicates threshold for epitope probability. Approximate locations of amino acid substitutions at residues 122, 215 and 217 are marked with arrows. Prediction was performed using the Bepipred 2.0 server [25].

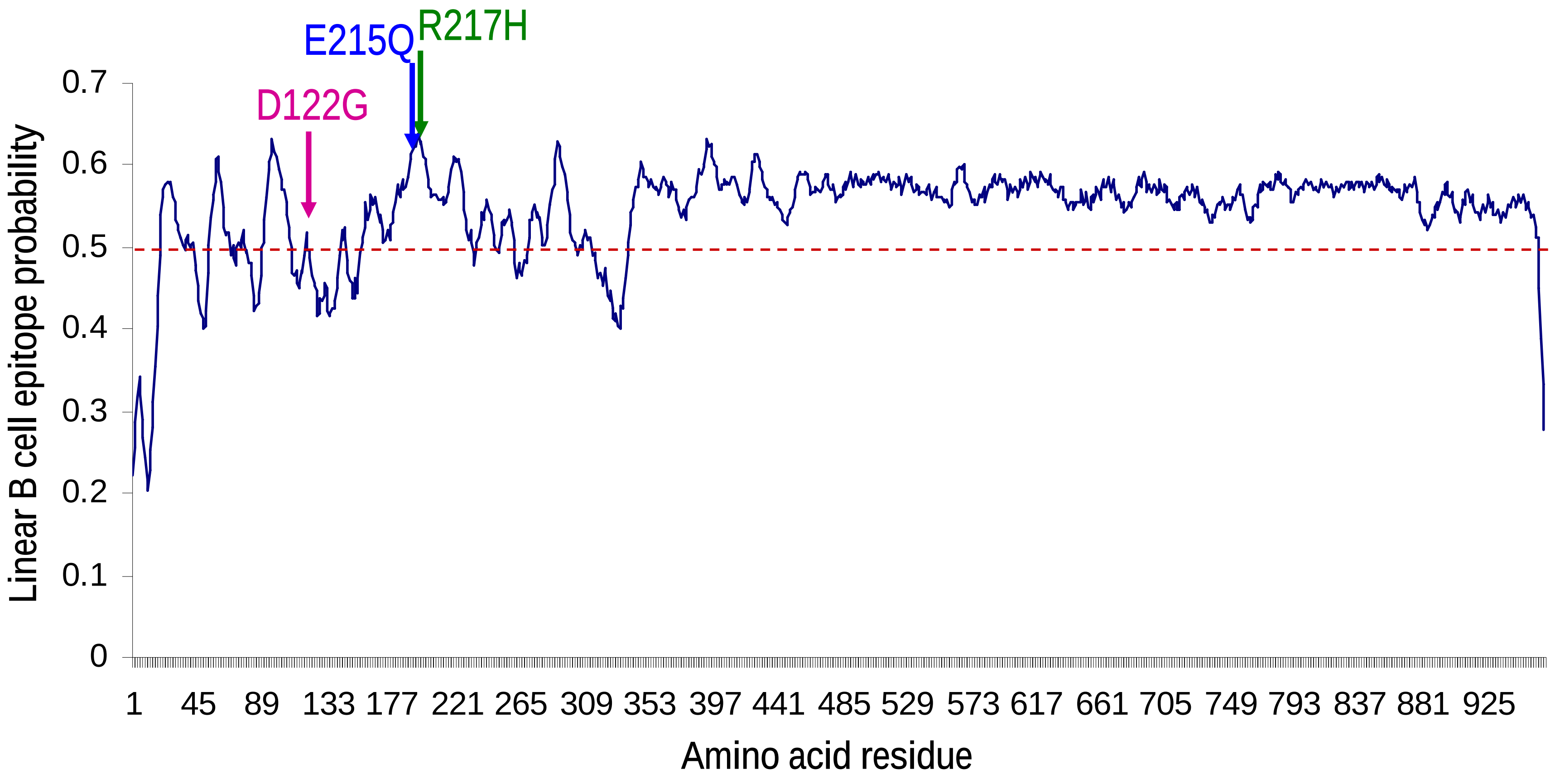

Supplement: Supplementary file 1 — Supplementary information 1. [file 41598_2021_82928_MOESM1_ESM.pdf]
